# Analysis of Different Feature Selection Criteria Based on a Covariance Convergence Perspective for a SLAM Algorithm

**DOI:** 10.3390/s110100062

**Published:** 2010-12-23

**Authors:** Fernando A. Auat Cheein, Ricardo Carelli

**Affiliations:** Instituto de Automatica, National University of San Juan, Av. Libertador Gral. San Martin 1109 Oeste, San Juan, Argentina; E-Mail: rcarelli@inaut.unsj.edu.ar

**Keywords:** SLAM, mapping, features selection

## Abstract

This paper introduces several non-arbitrary feature selection techniques for a Simultaneous Localization and Mapping (SLAM) algorithm. The feature selection criteria are based on the determination of the most significant features from a SLAM convergence perspective. The SLAM algorithm implemented in this work is a sequential EKF (Extended Kalman filter) SLAM. The feature selection criteria are applied on the correction stage of the SLAM algorithm, restricting it to correct the SLAM algorithm with the most significant features. This restriction also causes a decrement in the processing time of the SLAM. Several experiments with a mobile robot are shown in this work. The experiments concern the map reconstruction and a comparison between the different proposed techniques performance. The experiments were carried out at an outdoor environment composed by trees, although the results shown herein are not restricted to a special type of features.

## 1. Introduction

This paper addresses the problem of feature selection within a feature-based simultaneous localization and mapping (SLAM) algorithm. The feature selection methods shown herein are based on non-heuristic criteria in order to use only the most meaningful features according to the convergence theorem of the SLAM algorithm, in the correction stage of the SLAM.

The SLAM algorithm applied on a mobile robot recursively estimates the pose—localization and orientation—of the vehicle and the elements of the environment—called map—while reducing errors associated with the estimation process [1,2]. Several algorithms have been proposed as solutions to the SLAM problem. The most widely used by the scientific community is the Extended Kalman filter (EKF) [1,3–6] solution and its derived filters, such as the Unscented Kalman filter (UKF) [3] and the Extended Information filter (EIF) [7,8]. In these filters, the SLAM system state, composed by the robot’s pose and the map of the environment, it is modeled as a Gaussian random variable. Others solutions has also been implemented to solve the SLAM problem with high success, such as the case of the Particle filter (PF) [9,10], the *Graph-SLAM* [11,12] and the *FastSLAM* presented in [3,13].

Different SLAM algorithms solutions are presented to solve one or several issues associated with the SLAM process, such as the time consuming processing, the accuracy of the map, the successful closure of the loop, the integration of the SLAM algorithm with control laws to drive the vehicle motion and the modeling of different environments (dynamic, highly dynamic, static, structured, unstructured, *etc.*) [2,3,5]. Thus, for example, the EKF-SLAM presented in [4] map lines extracted from structured environments whereas in [14,15] works on environments with point-based features (parameterized as range and bearing). The EKF has also been used in vision-based SLAM. Despite the easy implementation of the EKF-SLAM, the correction part of it demands high computation resources. To solve this, the EIF is used instead of the EKF [3]. The PF arises as an improvement of the map accuracy and makes the SLAM process independent from the Gaussianity restriction of the EKF, although its real time implementation jointly with non-reactive control laws is still in development.

Several secondary process are involved within the SLAM algorithm, such as the case of the feature extraction process and the feature matching criterion. The feature extraction process determines the model associated with the environment and thus the map derived from the SLAM system state. The feature extraction procedure is also strongly related with the sensors incorporated on the mobile robot. Thus, for example, the line features or the point-based features mentioned before [4,14] are extracted by means of a range sensor laser, whereas the lines in [16] are extracted by a single camera. The feature extraction procedure is often a first environment filter of the SLAM. Those features whose quality is not acceptable for the mapping process or that have a certain probability of being a spurious measurement are rejected. The matching or data association is also crucial in the SLAM algorithm. A bad feature association could lead the SLAM to inconsistence [1,2]. Many feature association techniques have been proposed in the scientific literature, although the Mahalanobis distance is one of the most used criterion [3]. A successful matching will allow a successful SLAM.

This paper introduces several non-heuristic criteria to select the most significant features from the environment to be used in the correction stage of the SLAM algorithm. The SLAM algorithm is implemented on an EKF. The selection criteria are based on the convergence theorem of the SLAM, restricting the correction stage of the estimation process to those features that contribute the most to the convergence of the determinant of the covariance matrix of the SLAM system state. Thus, four methods are presented: a first approach based on covariance ratio, a second approach based on the sum of the eigenvalues associated with the correction stage of the SLAM algorithm, a third approach based on the maximum eigenvalue also associated with the correction stage of the EKF-SLAM and a fourth approach based on the covariance matrices associated with the features extracted during the feature extraction procedure. The optimization criteria and the corresponding algorithms for such feature selection procedures are also included in this work along with the appropriate extensions in the case that the covariance Joseph’s form were used instead of the classical EKF covariance updating procedure [17]. Furthermore, the proposals are compared with a SLAM algorithm with an entropy-based feature selection and the full sequential EKF-SLAM [3]. Several experimental results and performance comparisons are also included in this work, showing the advantages of implementing a non-heuristic features selection method in a SLAM algorithm. Although the feature selection criteria presented herein are not restricted to the type of features used within the SLAM, an EKF-SLAM with point-based features is used to show the performance of each proposal.

## 2. Related Work

The need of selecting the features to be used by the SLAM algorithm is present at every SLAM algorithm design. The most common criterion is to select the best features from the feature extraction stage. Thus, those features without the quality demanded by the implementation would be rejected, such is the case shown in [18], where features that are not good enough for the mapping purpose are considered as spurious measurements. For example, in [4], the lines whose lengths are below a certain threshold are not added to the SLAM system state nor considered in the updating stage.

Another example of the feature selection application within the SLAM algorithm is the one presented in [15,19]. When the SLAM is implemented on real time processes, the processing time becomes crucial for avoiding open loop situations [20]. According to this, for the purpose of reducing the processing time associated with the correction stage of the EKF-SLAM algorithm, a restriction is made on the number of features to be used during the updating. Thus, the work of [15,19] uses only a fixed number of features chosen regarding different criteria, such as proximity to the vehicle, smallest covariance associated with the extraction procedure or simply by the order in which the features were detected.

On the other hand, the work of [21] presents a new criterion of chosen features according to the information provided by them to the SLAM algorithm. In order to do so, the entropy of the covariance matrix of the SLAM system state attached to each observed feature is calculated. If the *information difference*—see Section 4.1—is over a certain threshold, then that feature will be used in the correction stage of the SLAM algorithm; otherwise it will be discarded. The main disadvantages of this method is the high computational time associated with the calculation of the determinant of the covariance matrix of the SLAM system state—which grows as the number of features increases—and the selection of the *information difference* threshold. This threshold represents a compromise in the design of the selection procedure.

## 3. Sequential EKF-SLAM Algorithm

The SLAM algorithm solved by an EKF is stated in Equation (1). All variables involved in the estimation process are considered as Gaussian random variables.

(1) $$\begin{array}{c} {\hat{\xi }}_{t}^{-}=f\left({\hat{\xi }}_{t},u_{t}\right)\\ P_{t}^{-}=A_{t}P_{t-1}A_{t}^{T}+W_{t}Q_{t-1}W_{t}^{T}\\ K_{t}=P_{t}^{-}H_{t}^{T}{\left(H_{t}P_{t}^{-}H_{t}^{T}+R_{t}\right)}^{-1}\\ {\hat{\xi }}_{t}={\hat{\xi }}_{t}^{-}+K_{t}\left(z_{t}-h\left({\hat{\xi }}_{t}^{-}\right)\right)\\ P_{t}=\left(I-K_{t}H_{t}\right)P_{t}^{-} \end{array}$$

In Equation (1), ${\hat{\xi }}_{t}^{-}$ is the predicted state of the system at time *t*; *u_t_* is the input control commands and *ξ̂_t_* is the corrected state at time *t; f* describes the motion of the elements of *ξ̂*. $P_{t}^{-}$ and *P_t_* are the predicted and corrected covariance matrices respectively at time *t; A_t_* is the Jacobian of *f* with respect to the SLAM system state and *Q_t_* is the covariance matrix of the noise associated to the process, whereas *W_t_* is its Jacobian matrix; *K_t_* is the Kalman gain at time *t; H_t_* is the Jacobian matrix of the measurement model (*h*) and *R_t_* is the covariance matrix of the actual measurement (*z_t_*). The term $\left(z_{t}-h\left({\hat{\xi }}_{t}^{-}\right)\right)$ is called the innovation vector [3] and takes place when the data association procedure has reached an appropriated matching between the observed feature and the predicted one $\left(h\left({\hat{\xi }}_{t}^{-}\right)\right)$. Both, the process model (*f*) and the observation model are non-linear expressions. Further information about the EKF-SLAM can be found in [22].

The sequential EKF-SLAM is based on the iterative calculation of the correction stage (SLAM system state and covariance matrix) for each feature with correct association—see [3]. The last statement implies that the Jacobian matrix of the measurement model and Kalman gain are sparse matrices, decreasing in that way the processing time during a correction iteration. Nevertheless, the prediction stage remains as stated in Equation (1).

The general form of the correction stage of the classical sequential EKF-SLAM algorithm [3] is summarized in Algorithm 1. Sentences (3) to (9) describe the *for*-loop of the correction stage of the algorithm. For every feature with correct association–sentence (2)—the *for*-loop is executed. Sentence (4) shows the Kalman gain calculation; sentence (5) is the correction of the SLAM system state whereas sentence (6) is the correction of the covariance matrix of the SLAM algorithm; in sentence (7), the current feature is deleted from the set of features with correct association (*M_t_*). In the next iteration, the next predicted SLAM system state and covariance matrix are the last corrected SLAM system state and covariance matrix respectively, as noted in sentence (8).

**Algorithm 1.** Algorithm of the Sequential EKF-SLAM.

1:	Let *N_t_* be set of the observed features
2:	Let *M_t_* ⊆ *N_t_* be the set of features with correct association
3:	**for***j* = 1 to ⧣ *M_t_***do**
4:	$K_{t,j}=P_{t,j}^{-}H_{t,j}^{T}{\left(H_{t,j}P_{t,j}^{-}H_{t,j}^{T}+R_{t,j}\right)}^{-1}$
5:	${\hat{\xi }}_{t,j}={\hat{\xi }}_{t,j}^{-}+K_{t,j}\left(z_{j}-h\left({\hat{\xi }}_{t}^{-}\right)\right)$
6:	$P_{t,j}=\left(I-K_{t,j}H_{t}\right)P_{t,j}^{-}$
7:	*M_t_* = *M_t_* – {*z_j_*}
8:	$P_{t,j}^{-}:=P_{t,j};{\hat{\xi }}_{t,j}^{-}={\hat{\xi }}_{t,j}$
9:	**end for**

## 4. Features Selection Criteria

By exploiting the sequentiality condition of the EKF-SLAM presented in Algorithm 1, the following sections will introduce several feature selection approaches for choosing the most significant features to be used in the correction stage of the SLAM from a non-arbitrary perspective.

Thus, Section 4.1 shows a method for selecting features of the environment by means of the entropy associated with them; Section 4.2 shows the feature selection criterion based on the covariance ratio of the SLAM algorithm; Section 4.3 shows two selection criteria based on the eigenvalues associated with the covariance ratio of the SLAM algorithm; Section 4.4 shows the modifications of the previous feature selection criteria when the Joseph’s form of the covariance matrix is used in the correction stage of the EKF-SLAM instead the one presented in Equation (1) and Section 4.5 shows the feature selection criteria based on covariance matrices associated with the features’ extraction procedure.

### 4.1. Features Selection: Entropy Approach

The SLAM algorithm with feature selection based on the observation of the entropy of the measurements was previously presented by [21]. This algorithm is considered as related to the proposal herein. This method is based on the calculation of the entropy attached to each observed feature. If the entropy is below a certain threshold value, then the observation will be computed in the correction stage of the EKF.

Considering that all variables involved in the EKF-SLAM estimation process are Gaussian random variables, the entropy value associated with a single observation can be represented as it is shown in Equation (2).

(2) $$\sum_{t}=E-\text{ln}p\left(\xi_{t}|z_{i}\right)=0.5\text{ln}\left[{\left(2\pi e\right)}^{n}\left|P_{t}\right|\right]$$

In Equation (2), ∑ is the entropy of the observation *z*. The *a priori* and *posteriori* information metric can be defined as the inverse of the entropy value, shown in Equation (2). Thus,

(3) $${\mathrm{im}}_{t}=-\sum_{t}=-0.5\text{ln}\left[{\left(2\pi e\right)}^{n}\left|P_{t}\right|\right]$$

(4) $${\mathrm{im}}_{t}^{-}=-\sum_{t}^{-}=-0.5\text{ln}\left[{\left(2\pi e\right)}^{n}\left|P_{t}^{-}\right|\right]$$

The information difference can be calculated as in Equation (5), where the absolute incremental information is obtained.

(5) $$\Delta_{i}={\mathrm{im}}_{t}-{\mathrm{im}}_{t}^{-}$$

Thus, when the absolute information of a feature exceeds a certain threshold (*δ*), that feature will be used in the correction stage of the EKF-SLAM algorithm. The algorithm of the EKF-SLAM with feature selection based on the entropy is summarized in Algorithm 2.

As Algorithm 2 shows, the calculation of the entropy associated with a single observation—and its information metric—is related to the determinant of the complete covariance matrix of the SLAM system state. Thus, the complexity of the calculation of the entropy is *O*(*n*^2^), where *n* is the dimension of the SLAM system state. Since this dimension varies, the complexity of the algorithm varies as well. Although this algorithm has the advantage of restricting the number of features to be updated, the calculation of the entropy requires the calculation of the determinant of the SLAM system state covariance matrix (*P_t_*), which in fact increases the processing time of the EKF-SLAM algorithm. Further details on this approach can be found in [21].

**Algorithm 2.** Algorithm of the EKF-SLAM based on the entropy feature selection procedure.

1:	Let *N_t_* be set of the observed features at time t
2:	Let *M_t_* ⊆ *N_t_* be the set of features with correct association at time t
3:	∀*z_i_* ∈ *M_t_*, *P_t_|z_i_* is calculated according to Equation (1)
4:	**if** Δ*_i_* ≥ *δ***then**
5:	Update equation $\left({\hat{\xi }}_{t}={\hat{\xi }}_{t}^{-}+K_{t}\left(z_{t}-h\left({\hat{\xi }}_{t}^{-}\right)\right)\right)$ takes place.
6:	**end if**

### 4.2. Features Selection: Covariance Ratio Approach

The covariance ratio approach as feature selection criterion in the EKF-SLAM algorithm was formerly published by the authors in [23]. This approach is based on the evaluation of the influence of a given feature—with correct association—in the convergence of the covariance matrix of the SLAM system state. The correction of the covariance matrix of the SLAM system state can be expressed as:

$$P_{t}=\left(I-K_{t}H_{t}\right)P_{t}^{-}=P_{t}^{-}-K_{t}H_{t}P_{t}^{-}$$

Applying the determinant to both sides of the expression above, it follows:

(6) $$\left|P_{t}\right|=\left|I-K_{t}H_{t}\right|\left|P_{t}^{-}\right|=\left|P_{t}^{-}-K_{t}H_{t}P_{t}^{-}\right|$$

Considering that in Equation (6), $P_{t}^{-}$ is *pd* (positive definite) and $K_{t}H_{t}P_{t}^{-}$ is *psd* (positive semi-definite) matrices, the above expression leads to Equation (7).

(7) $$\left|P_{t}\right|=\left|I-K_{t}H_{t}\right|\left|P_{t}^{-}\right|=\left|P_{t}^{-}-K_{t}H_{t}P_{t}^{-}\right|\leq \left|P_{t}^{-}\right|$$

Then, from Equations (6) and (7):

(8) $$0\leq \frac{\left|P_{t}\right|}{\left|P_{t}^{-}\right|}=\left|I-K_{t}H_{t}\right|\leq 1;\;\;\mathrm{with}\left|P_{t}^{-}\right|\neq 0$$

Equation (8) defines the covariance ratio of the SLAM algorithm. In this case, the ratio is used as a measure of the volume of the uncertainty ellipse associated with the covariance matrix of the SLAM system state [24].

Another convergence property states that, at the limit, all elements of *P_t_* become fully correlated [24]. This last statement is equivalent to say that,

(9) $$\underset{t\to \infty }{\text{lim}}\left|P_{t}\right|=0$$

Thus, according to Equations (8) and (9), given a set of observed features with correct matching, the feature that causes the highest decrease of |*P_t_*|, is the feature to which the EKF-SLAM is more sensitive to and will cause the fastest convergence of Equation (9). This latter point can be regarded as an optimization problem. Let *N_t_* be the set of observed features at time *t*; let *M_t_* ⊆ *N_t_* be set of features with correct association. Then ∀*z* ∈ *M_t_* ⊆ *N_t_*:

(10) $$z^{\mathrm{opt}}:\arg_{z}\min\left(|P_{t}|\right)\equiv \arg_{z}\min\left(|I-K_{t}H_{t}|\right)$$

Thus, according to Equation (10), finding the observation *z* that minimizes |*I – K_t_H_t_*| is equivalent to finding the observed feature that causes the highest decrease of |*P_t_*| because $\left|P_{t}^{-}\right|$ is independent of the current observation.

Considering that the EKF-SLAM implemented in this work is a sequential algorithm [3], the Jacobian of the observation model has the sparse form shown in Equation (11), where *H*_*v*,*t*_ is the Jacobian of the observation model with respect to the vehicle’s degrees of freedom and *H*_*z*,*t*_ is the Jacobian of the observation model with respect to the parameters of the observed feature. Θ_1_ and Θ_2_ are null matrices. The Kalman—Equation (12)—gain is also defined according to Equation (11).

(11) $$H_{t}=\left[\begin{array}{cccc} H_{v,t} & \Theta_{1} & H_{z,t} & \Theta_{2} \end{array}\right]$$

(12) $$K_{t}^{T}=\left[\begin{array}{cccc} K_{v,t}^{T} & K_{\Theta_{1}}^{T} & K_{z,t}^{T} & K_{\Theta_{2}}^{T} \end{array}\right]$$

Thus, the Jacobian of the observation is only calculated on the Jacobian entries that correspond to the vehicle and to the feature with correct association [1,2]. By using Equations (11) and (12), the determinant of |*I – K_t_H_t_*| can be calculated as:

(13) $$\begin{array}{c} \left|I-K_{t}H_{t}\right|=\\ =\left|\left[\begin{array}{cccc} I_{v} & & & \\ & I_{\Theta_{1}} & & \\ & & I_{z} & \\ & & & I_{\Theta_{2}} \end{array}\right]-\left[\begin{array}{c} K_{v,t}\\ K_{\Theta_{1}}\\ K_{z,t}\\ K_{\Theta_{2}} \end{array}\right]\left[\begin{array}{cccc} H_{v,t} & \Theta_{1} & H_{z,t} & \Theta_{2} \end{array}\right]\right|\\ =\left|\begin{array}{cccc} I_{v}-K_{v,t}H_{v,t} & \Theta & -K_{v,t}H_{z,t} & \Theta \\ -K_{\Theta_{1}}H_{v,t} & I_{\Theta_{1}} & -K_{\Theta_{1}}H_{z,t} & \Theta \\ -k_{z,t}H_{z,t} & \Theta & I_{z}-K_{z,t}H_{z,t} & \Theta \\ -K_{\Theta_{2}}H_{v,t} & \Theta & -K_{\Theta_{2}}H_{z,t} & I_{\Theta_{2}} \end{array}\right|\\ =|\begin{array}{cc} I_{v}-K_{v,t}H_{v,t} & -K_{v,t}H_{z,t}\\ -K_{z,t}H_{v,t} & I_{z}-K_{z,t}H_{z,t} \end{array}| \end{array}$$

In Equation (13)*I* is the identity matrix, *I_v_*, *I*_Θ_1__, *I_z_* and *I*_Θ_2__ are identity block matrices with the dimensions of *K_v,t_H_v,t_*, *K*_Θ_1__ Θ_1_, *K_z,t_H_z,t_* and *K*_Θ_2__ Θ_2_ respectively. If we consider that the vehicle has three degrees of freedom—two related to the position and one to the orientation—and the feature is determined by two parameters, then the final calculation of Equation (13) is a 5 *×* 5 matrix.

The correction stage of the EKF-SLAM algorithm with the feature selection based on the covariance ratio is presented in Algorithm 3.

**Algorithm 3.** Algorithm of the EKF-SLAM based on the covariance ratio feature selection procedure.

1:	Let *N_t_* be set of the observed features at time t
2:	Let *M_t_* ⊆ *N_t_* be the set of features with correct association at time t
3:	Let *LIM* be the maximum number of features to be used in the correction stage
4:	**for***j* = 1 to *min*{*LIM*, ⧣*M_t_*} **do**
5:	find $z_{j}^{\mathrm{opt}}$: *arg_z_min*(|*P_t,j_*|) ≡ *arg_z_min*(|*I* − *K_t,j_H_t,j_*|)
6:	$K_{t,j}=P_{t,j}^{-}H_{t,j}^{T}{\left(H_{t,j}P_{t,j}^{-}H_{t,j}^{T}+R_{t,j}\right)}^{-1}|z_{j}^{\mathrm{opt}}$
7:	${\hat{\xi }}_{t,j}={\hat{\xi }}_{t,j}^{-}+K_{t,j}\left(z_{j}^{\mathrm{opt}}-h\left({\hat{\xi }}_{t,j}^{-}\right)\right)$
8:	$P_{t,j}=(I-K_{t,j}H_{t,j}))P_{t}^{-}|z_{j}^{\mathrm{opt}}$
9:	$M_{t}=M_{t}-\{z_{j}^{\mathrm{opt}}\}$
10:	$P_{t,j}^{-}:=P_{t,j};{\hat{\xi }}_{t,j}^{-}={\hat{\xi }}_{t,j}$
11:	**end for**

In Algorithm 3, sentences (1) − (2) are the declaration of the domain that is going to be used in the correction stage; sentence (3) determines—if possible—the maximum number of features that will be used for correcting the SLAM. If the number of features in *M_t_* is smaller than *LIM*, then the complete set of features in *M_t_* will be used in the correction loop. Sentences (4) − (9) show the *for*-loop of the correction stage. Given *M_t_*, the algorithm searches for a first *z^opt^*. When it is found, the correction takes place—(6) to (8)—and this features is removed from *M_t_*. In the second iteration of the *for*-loop, the *z^opt^* is searched inside the new *M_t_* and both the actual predicted system state and covariance are the last corrected system state and covariance matrix as shown in sentence (10). This situation ensures that sequentiality of the EKF-SLAM is not lost.

### 4.3. Features Selection: Eigenvalues Approach

In this approach, instead of selecting the features according to the determinant of Equation (13), the eigenvalues associated with it will be used.

By inspection, it is possible to see that if an eigenvalue of Equation (13) tends to zero faster than the others, then that eigenvalue will dominate the convergence of |*P_t_*|—see Equation (9). Thus, the eigenvalues approaches presented herein give a better description of the behavior of the set of eigenvalues associated with (*I − K_t_H_t_*) in Equation (13), because they consider the behavior of all eigenvalues.

Let us calculate the eigenvalues of Equation (13)—*Eig*(*I − K_t_H_t_*). Applying the definition of eigenvalues we have that:

(14) $$\begin{array}{c} \left|\left(I-K_{t}H_{t}\right)-\lambda I\right|=\\ \left|\begin{array}{cccc} A_{1,1} & \Theta & A_{1,3} & \Theta \\ A_{2,1} & A_{2,2} & A_{2,3} & \Theta \\ A_{3,1} & \Theta & A_{3,3} & \Theta \\ A_{4,1} & \Theta & A_{4,3} & A_{4,4} \end{array}\right| \end{array}$$

with,

$$\begin{array}{l} A_{1,1}=I_{v}-K_{v,t}H_{v,t}-\lambda I_{v}\\ A_{1,3}=-K_{v,t}Hz,t\\ A_{2,1}=-K_{\Theta_{1}}H_{v,t}\\ A_{2,2}=I_{\Theta_{1}}-\lambda I_{\Theta_{1}}\\ A_{2,3}=-K_{\Theta_{1}}H_{z,t}\\ A_{3,1}=-K_{z,t}H_{z,t}\\ A_{3,3}=I_{z}-K_{z,t}H_{z,t}-\lambda I_{z}\\ A_{4,1}=-K_{\Theta_{2}}H_{v,t}\\ A_{4,3}=-K_{\Theta_{2}}H_{z,t}\\ A_{4,4}=I_{\Theta_{2}}-\lambda I_{\Theta_{2}}\\ \Theta \;\text{is a null matrix with the appropriate dimensions} \end{array}$$

By inspection of Equation (14) and considering that Θ is a null matrix with the appropriate dimension, it is possible to see that,

(15) $$\begin{array}{c} \left|\left(I-K_{t}H_{t}\right)-\lambda I\right|=\\ {\left(1-\lambda \right)}^{r}\left|\begin{array}{cc} I_{v}-K_{v,t}H_{v,t}-\lambda I_{v} & -K_{v,t}H_{z,t}\\ -K_{z,t}H_{v,t} & I_{z}-K_{z,t}H_{z,t}-\lambda I_{z} \end{array}\right| \end{array}$$

Thus, the only eigenvalues of (*I − K_t_H_t_*) affected by the current feature *z_i_* are the eigenvalues of $\left[\begin{array}{cc} I_{v}-K_{v,t}H_{v,t} & -K_{v,t}H_{z,t}\\ -K_{z,t}H_{v,t} & I_{z}-K_{z,t}H_{z,t} \end{array}\right]$. The rest of the eigenvalues equal one—see Equation (15). Considering that the pose of the robot has three degrees of freedom—two associated with the position and one with the orientation—and the feature has 2 parameters that define it, then the calculation of the eigenvalues of (*I − K_t_H_t_*)—which is an *n × n* matrix—is reduced to the calculation of a 5 *×* 5 matrix.

In this section, two eigenvalues approaches are presented for selecting features. The first approach consists on choosing the features according with the sum of eigenvalues of Equation (15). Thus, from the set *M_t_* of features with appropriate association, only the feature with the minimum sum of its eigenvalues will be selected.

The other approach is to select the features based on the lowest value of the highest eigenvalue. Equation (16) shows the selection criterion based on the sum of eigenvalues whereas Equation (17) shows the selection criterion based on the value of the highest eigenvalue associated with a feature.

(16) $$\begin{array}{c} \forall z_{i}\in M_{t},z^{\mathrm{opt}}\equiv \arg_{z}\min\left(\left|P_{t}\right|\right)\equiv \\ \arg_{z}\min(\sum \mathrm{Eig}(\left[\begin{array}{cc} I_{v}-K_{v,t}H_{v,t} & -K_{v,t}H_{z,t}\\ -K_{z,t}H_{v,t} & I_{z}-K_{z,t}H_{z,t} \end{array}\right])) \end{array}$$

(17) $$\begin{array}{c} \forall z_{i}\in M_{t},z^{\mathrm{opt}}\equiv \arg_{z}\min\left(\left|P_{t}\right|\right)\equiv \\ \arg_{z}\min(\mathrm{MAX}\;\mathrm{Eig}(\left[\begin{array}{cc} I_{v}-K_{v,t}H_{v,t} & -K_{v,t}H_{z,t}\\ -K_{z,t}H_{v,t} & I_{z}-K_{z,t}H_{z,t} \end{array}\right])) \end{array}$$

**Algorithm 4.** Algorithm of the EKF-SLAM based on the eigenvalues selection approach.

1:	Let *N_t_* be set of the observed features at time t
2:	Let *M_t_* ⊆ *N_t_* be the set of features with correct association at time t
3:	Let *LIM* be the maximum number of features to be used in the correction stage
4:	**for***j* = 1 to *min*{*LIM*, ⧣*M_t_*} **do**
5:	find $z_{j}^{\mathrm{opt}}$: *arg_z_min*(|*P_t_*,*_j_*|)
6:	$K_{t,j}=P_{t,j}^{-}H_{t,j}^{T}{\left(H_{t,j}P_{t,j}^{-}H_{t,j}^{T}+R_{t,j}\right)}^{-1}|z_{j}^{\mathrm{opt}}$
7:	${\hat{\xi }}_{t,j}={\hat{\xi }}_{t,j}^{-}+K_{t,j}\left(z_{j}^{\mathrm{opt}}-h\left({\hat{\xi }}_{t,j}^{-}\right)\right)$
8:	$P_{t,j}=(I-K_{t,j}H_{t,j}))P_{t,j}^{-}|z_{j}^{\mathrm{opt}}$
9:	$M_{t}=M_{t}-\{z_{t,j}^{\mathrm{opt}}\}$
10:	$P_{t,j}^{-}:=P_{t,j};{\hat{\xi }}_{t,j}^{-}={\hat{\xi }}_{t,j}$
11:	**end for**

Thus, in Equation (16), the feature selected has the minimum sum of eigenvalues; in Equation (17), the feature selected is the one which has the smallest maximum eigenvalue. The last is based on that if the higher eigenvalue decreases, also decrease (or remain equal) the rest of the eigenvalues. Thus, this method allows a selection of features based on the behavior of the eigenvalues. Further information about the *sum of eigenvalues* method can be found in [20].

Algorithm 4 shows the general structure of the selection procedure. Sentence (5) can be chose according to Equations (16) or (17).

### 4.4. Features Selection: Joseph’s Covariance Matrix Approach

Up to now, the feature selection approaches presented are based on the covariance matrix of the SLAM system state: $P_{t}=\left(I-K_{t}H_{t}\right)P_{t}^{-}$. Due to the fact of possible lost of positive definiteness of *P_t_* during the numerical computation, the Joseph’s form of the covariance matrix of the SLAM system state within an EKF-SLAM is widely used by the scientific community [17]. The Joseph’s form is shown in Equation (18).

(18) $$P_{t}=\left(I-K_{t}H_{t}\right)P_{t}^{-}{\left(I-K_{t}H_{t}\right)}^{T}+K_{t}R_{t}K_{t}^{T}$$

In Equation (18)*R_t_* is the covariance matrix of the observation. As it can be noted, the expression above corresponds to an *n × n* matrix, where *n* is the order of the SLAM system state.

In order to reduce the computational cost by applying any selection criterion previously presented with Equation (18) instead of Equation (1) some calculations are needed.

Thus, considering that $\left(I-K-tH_{t}\right)P_{t}^{-}{\left(I-K_{t}H_{t}\right)}^{T}$ and $K_{t}R_{t}K_{t}^{T}$ in Equation (18) are *psd* the two following conditions hold.

(19) $$\{\begin{array}{l} \left|P_{t}\right|\geq \left|\left(I-K_{t}H_{t}\right)P_{t}^{-}{\left(I-K_{t}H_{t}\right)}^{-}\right|\\ ={\left|I-K_{t}H_{t}\right|}^{2}\left|P_{t}\right|\\ \left|P_{t}\right|\geq \left|K_{t}R_{t}K_{t}^{T}\right| \end{array}$$

In Equation (19), $\left|P_{t}^{-}\right|$ is a constant value because $P_{t}^{-}$ is independent of the current feature; in addition, in Equation (19) |*I − K_t_H_t_*|^2^ ≤ |*I − K_t_H_t_*| ≤ 1 according to Equation (8).

The calculation of *|I − K_t_H_t_|* applies as was shown in Equation (13). On the other hand, considering that $K_{t}R_{t}K_{t}^{T}=P_{t}^{-}H_{t}^{T}\Psi_{t}^{-1}R_{t}\Psi_{t}^{-T}H_{t}P_{t}^{-T}$, with $\Psi_{t}=H_{t}P_{t}^{-}H_{t}^{T}+R_{t}$, then, it follows that $\left|K_{t}R_{t}K_{t}^{T}\right|={\left|P_{t}^{-}\right|}^{2}\left|H_{t}^{T}\Psi_{t}^{-1}R_{t}\Psi_{t}^{-T}H_{t}\right|$. Replacing $M_{t}=\Psi_{t}^{-1}R_{t}\Psi_{t}^{-T}$, where *M* has the dimensions of *R_t_* −2 × 2 matrix, then,

$$\left|K_{t}R_{t}K_{t}^{T}\right|={\left|P_{t}^{-}\right|}^{2}\left|H_{t}^{T}M_{t}H_{t}\right|$$

Considering also that the EKF-SLAM used in this work is a sequential EKF, the above expression can be written as it is shown in Equation (20).

(20) $$\begin{array}{c} H_{t}^{T}M_{t}H_{t}=\\ ={\left[\begin{array}{cccc} H_{v,t}^{T} & \Theta_{1}^{T} & H_{z,t}^{T} & \Theta_{2}^{T} \end{array}\right]}^{T}M_{t}\left[\begin{array}{cccc} H_{v,t} & \Theta_{1} & H_{z,t} & \Theta_{2} \end{array}\right]\\ =\left[\begin{array}{cccc} H_{v,t}^{T}M_{t}H_{v,t} & \Theta & H_{v,t}^{T}M_{t}H_{z,t} & \Theta \\ \Theta & \Theta & \Theta & \Theta \\ H_{z,t}^{T}M_{t}H_{v,t} & \Theta & H_{z,t}^{T}M_{t}H_{z,t} & \Theta \\ \Theta & \Theta & \Theta & \Theta \end{array}\right] \end{array}$$

In Equation (20), Θ means a null block matrix with the appropriate dimensions. By inspection of Equation (20) is possible to see that $\left|P_{t}^{-2}\right|\left|H_{t}^{T}M_{t}H_{t}\right|=\left|K_{t}R_{t}K_{t}^{T}\right|=0$ which leads to the following expressions.

(21) $$\begin{array}{c} \left|P_{t}^{-}\right|{\left|I-K_{t}H_{t}\right|}^{2}\leq \left|P_{t}\right|\leq \left|P_{t}^{-}\right|\\ {\left|I-K_{t}H_{t}\right|}^{2}\leq \frac{\left|P_{t}\right|}{\left|P_{t}^{-}\right|}\leq 1;\;\;\mathrm{with}\left|P_{t}^{-}\right|>0 \end{array}$$

Thus, as it can be seen in Equation (21), smaller the determinant of |*I* – *K_t_H_t_*|, smaller the value that |*P_t_*| could adopt. Re-writing Equation (21) follows that,

(22) $$1\leq \frac{\left|P_{t}^{-}\right|}{\left|P_{t}\right|}\leq \frac{1}{{\left|I-K_{t}H_{t}\right|}^{2}}$$

Equation (22) implies that the smaller *|I − K_t_H_t_|* the bigger the inverse of the covariance ratio presented in Equation (8). Concluding, for a covariance matrix of the EKF-SLAM system state corrected according to the Joseph’s form, the feature selection criterion is the same as the one presented in Equation (10): *z^opt^* : *arg_z_min*(*|P_t_|*) ≡ *arg_z_min*(*|I − K_t_H_t_|*). From this last statement is possible to see that the feature selection approaches presented in Sections 4.2 and 4.3 apply in the same way when the Joseph’s form of the covariance correction is used.

### 4.5. Features Selection: Features’ Covariance Approach

**Figure 1.**
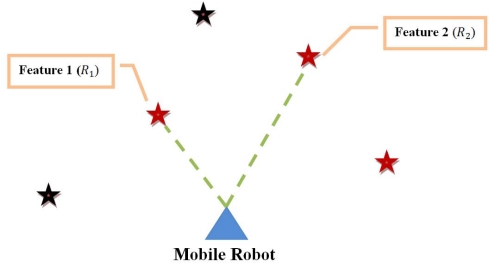
Graphic representation of two features with correct association (in red-star) among a set of five features extracted from the environment by the mobile robot’s sensors.

The *features’ covariance* approach is based on the following assumption. Let us suppose that at a time instant *t*, the mobile robot extracts five features from the environment (⧣*N_t_* = 5 in Algorithm 1). From the set of five features extracted, only two of them have appropriate association with the predicted features from the SLAM system state (⧣*M_t_* = 2 *<* ⧣*N_t_* in Algorithm 1). Figure 1 shows this situation. Each of these two features has associated a covariance matrix (*R*_1_ and *R*_2_ respectively) which intrinsically depends on the feature extraction procedure. Also, *R*_1_ and *R*_2_ are positive definite matrices. The *features’ covariance* approach consists in choosing the feature with the *smallest* covariance matrix with respect to the covariance matrices of the rest of features with appropriate association. Thus, for example, in Figure 1, if *R*_1_ is *smaller* than *R*_2_—(*R*_2_ − *R*_1_) is positive semi-definite, then *Feature 1* will be used within the correction stage of the EKF-SLAM instead of *Feature 2*. Although it seems intuitive to choose *R*_1_—because of its smaller covariance, the following theorem is the corresponding mathematical justification of the *features’ covariance* approach criterion.

**Theorem**—Let *R*_1_ and *R*_2_ be two symmetric positive definite covariance matrices associated with two features from the environment with correct association—as it is shown in Figure 1. Also, let *R*_2_ ≽ *R*_1_ (≽ stands for *positive semi-definite*, therefore, *R*_2_ *− R*_1_ ≽ 0), then,

(23) $$\left|P_{t}^{R_{1}}\right|\leq \left|P_{t}^{R_{2}}\right|.-$$

The theorem above establishes that the feature with associated covariance matrix *R*_1_ will cause the highest decrement of the uncertainty volume of the covariance matrix of the EKF-SLAM, *|P_t_|*, when compared with *R*_2_—see Equation (9).

**Proof**—By hypothesis, we have that:

(24) $$R_{1}\;≼\;R_{2}$$

Considering that $H_{t}P_{t}^{-}H_{t}^{T}$ in Equation (1) is positive semi-definite, then the above relation does not change if we add $H_{t}P_{t}^{-}H_{t}^{T}$ on both members of Equation (24).

(25) $$H_{t}P_{t}^{-}H_{t}^{T}+R_{1}\;≼\;H_{t}P_{t}^{-}H_{t}^{T}+R_{2}$$

Given that *R*_1_ and *R*_2_ are positive definite (by hypothesis), then the matrix $H_{t}P_{t}^{-}H_{t}^{T}+R_{i}$, with *i* = 1, 2, is also positive definite. Therefore, its inverse exists. Then,

(26) $${\left(H_{t}P_{t}^{-}H_{t}^{T}+R_{1}\right)}^{-1}≽{\left(H_{t}P_{t}^{-}H_{t}^{T}+R_{2}\right)}^{-1}$$

Equation (26) shows that, after applying the inverse at both sides of Equation (25) the positive definition between matrices changes [25]. Rewriting Equation (26) and pre-multiplying by $P_{t}^{-}H_{t}^{T}$ and post-multiplying by $H_{t}P_{t}^{-}$ respectively—observe that $P_{t}^{-}H_{t}^{T}={\left(H_{t}P_{t}^{-}\right)}^{T}$—we have that,

(27) $$\begin{array}{c} P_{t}^{-}H_{t}^{T}\left({\left(H_{t}P_{t}^{-}H_{t}^{T}+R_{1}\right)}^{-1}-{\left(H_{t}P_{t}^{-}H_{t}^{T}+R_{1}+R_{2}\right)}^{-1}\right)H_{t}P_{t}^{-}\;≽\;0\\ P_{t}^{-}H_{t}^{T}{\left(H_{t}P_{t}^{-}H_{t}^{T}+R_{1}\right)}^{-1}H_{t}P_{t}^{-}-P_{t}^{-}H_{t}^{T}{\left(H_{t}P_{t}^{-}H_{t}^{T}+R_{2}\right)}^{-1}H_{t}P_{t}^{-}\;≽\;0 \end{array}$$

Observe that the matrices definition between Equations (26) and (27) is not lost (see [25]). Finally, adding and subtracting $P_{t}^{-}$ in Equation (27) we have the following result.

(28) $$\left(P_{t}^{-}-P_{t}^{-}H_{t}^{T}{\left(H_{t}P_{t}^{-}H_{t}^{T}+R_{2}\right)}^{-1}H_{t}P_{t}^{-}\right)-\left(P_{t}^{-}-P_{t}^{-}H_{t}^{T}{\left(H_{t}P_{t}^{-}H_{t}^{T}+R_{1}\right)}^{-1}H_{t}P_{t}^{-}\right)\;≽\;0$$

Thus, according to Equations (1, 28) implies that,

$$P_{t}^{R_{2}}-P_{t}^{R_{1}}\;≽\;0$$

where $P_{t}^{R_{i}}$ is the covariance matrix of the SLAM algorithm correction stage if the feature with covariance matrix *R_i_* were used (*i* = 1, 2).

Considering that both $P_{t}^{R_{2}}$ and $P_{t}^{R_{1}}$ are positive definite and $P_{t}^{R_{2}}-P_{t}^{R_{1}}\;≽\;0$, then, according to [25], $\left|P_{t}^{R_{2}}\right|\geq \left|P_{t}^{R_{1}}\right|$. Therefore, having into account Equation (9), we can conclude that the feature that has the covariance matrix *R*_1_ associated with it will cause the highest decrement in the determinant of the covariance matrix of the SLAM system state correction stage. Then, that feature is the most meaningful from the convergence perspective of the SLAM algorithm.

The Algorithm 5 presents the *Features’ Covariance* approach for selecting the most meaningful features. The code line (6) shows the implementation of the selection criterion based on the covariance matrix of the features with correct association. In the case that a *z^opt^* is not found, then the correction of the EKF-SLAM is performed based on the detected features with correct association. Thus, *i.e.*, if *LIM* = 2 and ⧣*M_t_* ≥ 2 in the Algorithm 5 and no *z^opt^* exists, then the correction is performed with any two features from *M_t_*—code line (2) and (3).

## 5. Experimental Results

**Figure 2.**
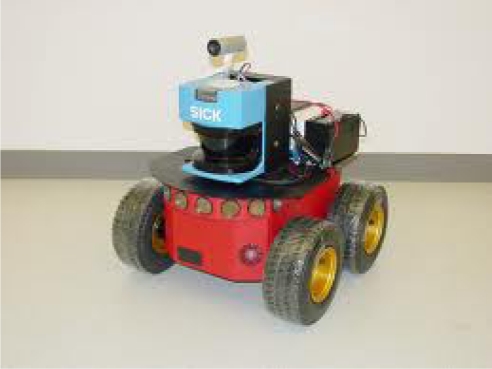
Picture of the mobile robot Pioneer 3AT used in this work. The range sensor laser is mounted on the vehicle.

The mobile robot used in this work is a nonholonomic unicycle type Pioneer 3AT built by **ActivMedia** with a range sensor laser SICK incorporated on it. The laser acquires 181 measurements in a range of 30 meters, from 0 to 180 degrees. Figure 2 shows the mobile robot used as well as the SICK laser mounted on it.

The feature extraction procedure was based on a clustering algorithm to extract point-based features, as the one shown in [14]. The parameters of the features were their range and bearing. The experiment were carried out outdoors and each detected feature was associated with a tree of the environment. The SLAM system state was composed by both: the vehicle degrees of freedom (position and orientation) and the parameters of the features according with their extraction instant. Further and detailed information about the SLAM initialization, feature extraction and implementation issues can be found in [1,3,14].

**Algorithm 5.** Algorithm of the EKF-SLAM based on the *features’ covariance* selection procedure.

1:	Let *N_t_* be set of the observed features at time t
2:	Let *M_t_* ⊆ *N_t_* be the set of features with correct association at time t
3:	Let *LIM* be the maximum number of features to be used in the correction stage
4:	**for***j* = 1 to *min*{*LIM*, ⧣*M_t_*} **do**
5:	*z_j_*, *R_t,j_*
6:	find $z_{j}^{\mathrm{opt}}$: *arg_z_min*(|*P_t,j_*|) ≡ *arg_z_min*((*R_t_*,*_j_*))
7:	$K_{t,j}=P_{t,j}^{-}H_{t,j}^{T}{\left(H_{t,j}P_{t,j}^{-}H_{t,j}^{T}+R_{t,j}\right)}^{-1}|z_{j}^{\mathrm{opt}}$
8:	${\hat{\xi }}_{t,j}={\hat{\xi }}_{t,j}^{-}+K_{t,j}\left(z_{j}^{\mathrm{opt}}-h\left({\hat{\xi }}_{t,j}^{-}\right)\right)$
9:	$P_{t,j}=(I-K_{t,j}H_{t,j}))P_{t}^{-}|z_{j}^{\mathrm{opt}}$
10:	$M_{t}=M_{t}-\{z_{j}^{\mathrm{opt}}\}$
11:	$P_{t,j}^{-}:=P_{t,j};{\hat{\xi }}_{t,j}^{-}={\hat{\xi }}_{t,j}$
12:	**end for**

For the purpose of testing the algorithms proposed in Section 4 and see their differences and advantages, the following considerations must be taken into account:

- The robot should navigate within the same environment
- The robot should follow the same path within the environment in order to ensure that each SLAM algorithm with the corresponding feature selection criterion visits the same zones.

**Figure 3.**
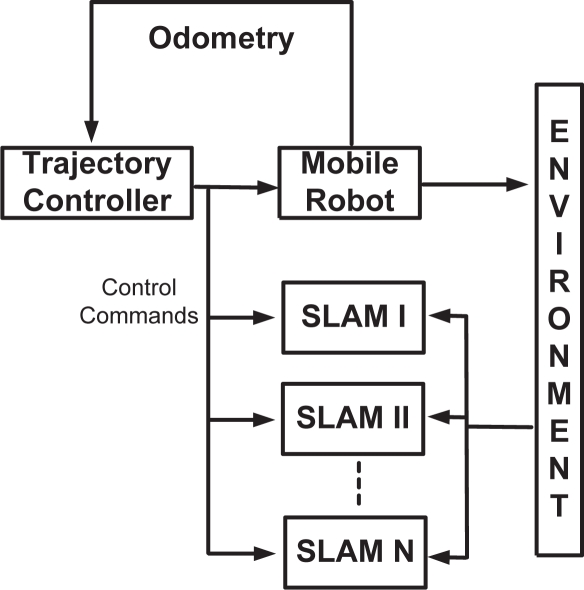
General architecture of the system. The trajectory controller uses the odometry of the mobile robot as input. The SLAM algorithms work independently from each other and from the controller.

In order to achieve such conditions, all the SLAM algorithms were implemented in parallel. The mobile robot followed a pre-established path by means of the Kanayama’s trajectory controller [26]. The path was previously determined by a differential GPS (built by **Novatel**). Also, the positions of the trees within the environment were previously measured by the differential GPS. Considering that the differential GPS measurement had an absolute error of ±0.1 meters, those measurements were used to compare the SLAM localization and mapping results. The mobile robot pose information provided to the controller was obtained from the fusion of odometry information with the differential GPS information (improving the odometry of the vehicle). Thus, no feedback is presented between the SLAM algorithms and the robot. Figure 3 shows the general architecture of the implemented system.

In this work, six different SLAM algorithms were implemented: the sequential EKF-SLAM shown in Algorithm 1, the entropy selection approach (see Algorithm 2), the covariance ratio approach (see Algorithm 3), the two feature selection eigenvalues approaches (see Algorithm 4 in Section 4.3) and the features’ covariance approach (see Algorithm 5).

### 5.1. SLAM Results

**Figure 4.**
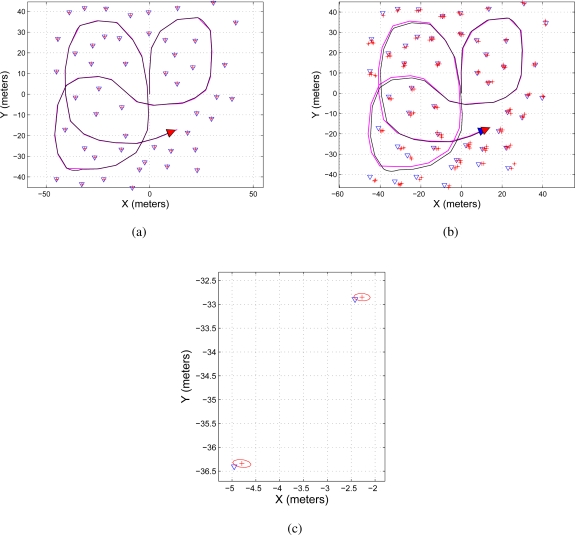
Map reconstruction using the sequential EKF-SLAM presented in Algorithm 1—without feature restriction. **(a)** Shows both: the map and the traveled path using the information processed by the sequential EKF-SLAM without restricting the number of features to be updated; **(b)** shows the sequential EKF-SLAM results after restricting the number of features to be updated to the first two features in being detected by the system; **(c)** it is a zoom to show the covariance ellipse associated with each feature of the environment. The path traveled by the mobile robot is represented in a solid black line and the path estimated by the SLAM in solid magenta; the features are represented by blue triangles and the ellipses of uncertainty associated with each feature are in solid red.

Figure 4 shows two maps representation of the environment. Figure 4(a) shows the map reconstruction when there is no restriction on the number of features to be used during the update stage of the EKF-SLAM; on the other hand, Figure 4(b) shows the map reconstruction when the sequential EKF-SLAM updating stage is restricted to the two first detected features; Figure 4(c) shows a zoom of Figure 4(a) where it can be seen that each feature has associated a covariance ellipse. The features of the environment are represented by blue triangles; the path traveled by the robot is a solid black line and the path estimated by the SLAM is a solid magenta line.

**Figure 5.**
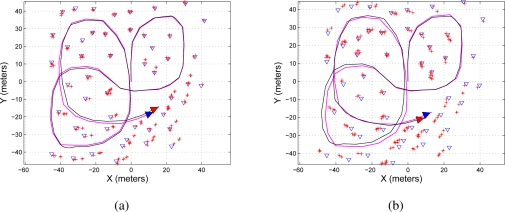
Map reconstruction of the environment using the entropy feature selection criterion on the EKF-SLAM. **(a)** Shows the map reconstruction when the information difference gate was set to *δ* = 0.2; **(b)** shows the map reconstruction when the information difference gate was set to *δ* = 0.4. As it can be seen, the map precision depends on the selection of the information difference gate, which in turn is related to the design of the algorithm. The path traveled by the mobile robot is represented in a solid black line and the path estimated by the SLAM in solid magenta; the features are represented by blue triangles and the ellipses of uncertainty associated with each feature are in solid red.

Figure 5 shows the map reconstruction of the environment based on the information provided by EKF-SLAM algorithm with entropy feature selection criterion shown in Algorithm 2. Figure 5(a) shows the map reconstruction when gate of the information difference was set to *δ* = 0.2; on the other hand, Figure 5(b) shows the map reconstruction for a gate of the information difference of *δ* = 0.4 (see the Algorithm 2). As it can be seen, the map reconstruction in Figure 5(b) is less precise than the one shown in Figure 5(a).

**Figure 6.**
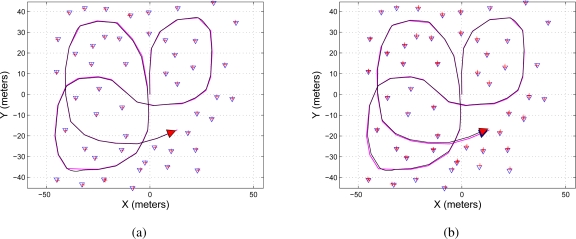
Map reconstruction of the environment using the covariance ratio feature selection criterion on the EKF-SLAM. **(a)** Shows the map reconstruction when the five most significant features —*LIM* = 5 in Algorithm 3—were used for the correction stage of the EKF-SLAM; **(b)** shows the map reconstruction when *LIM* = 2. The path traveled by the mobile robot is represented in a solid black line and the path estimated by the SLAM in solid magenta; the features are represented by blue triangles and the ellipses of uncertainty associated with each feature are in solid red.

The EKF-SLAM results based on the covariance ratio approach shown in Algorithm 3 are shown in Figure 6. In Figure 6(a), *LIM*—the maximum number of features to be selected—is set to *LIM* = 5. The last means that only the five most significant features from the covariance ratio approach point of view will be selected for the correction stage of the EKF-SLAM. Figure 6(b) shows the case when *LIM* = 2.

As it can be seen, the map constructed by the EKF-SLAM with the feature selection based on the covariance ratio approach (see Figure 6) is more similar to the one shown in Figure 4(a) than the map constructed by the EKF-SLAM with the entropy feature selection criterion (see Figure 5).

**Figure 7.**
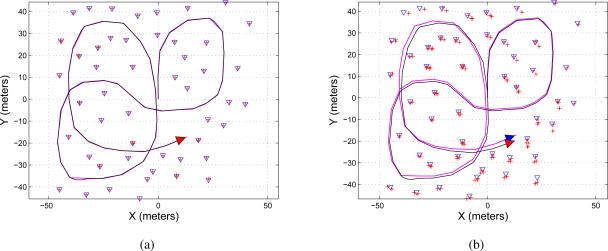
Map reconstruction of the environment using the sum of eigenvalues feature selection criterion on the EKF-SLAM. **(a)** Shows the map reconstruction when the five most significant features—*LIM* = 5 in Algorithm 4—were used for the correction stage of the EKF-SLAM; **(b)** shows the map reconstruction when *LIM* = 2. The path traveled by the mobile robot is represented in a solid black line and the path estimated by the SLAM in solid magenta; the features are represented by blue triangles and the ellipses of uncertainty associated with each feature are in solid red.

Figure 7 shows the map reconstruction based on the sum of eigenvalues feature selection criterion (shown in Algorithm 4). Figure 7(a) shows the case when *LIM* = 5 (the five most significant features are used in the correction stage of the EKF-SLAM); on the hand, in Figure 7(b), *LIM* = 2.

As it can be seen, Figure 7(a) is very similar to Figure 4(a).

**Figure 8.**
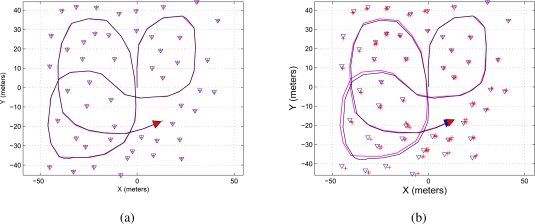
Map reconstruction of the environment using the maximum eigenvalue feature selection criterion on the EKF-SLAM. **(a)** Shows the map reconstruction when the five most significant features—*LIM* = 5 in Algorithm 4—were used for the correction stage of the EKF-SLAM; **(b)** shows the map reconstruction when *LIM* = 2. The path traveled by the mobile robot is represented in a solid black line and the path estimated by the SLAM in solid magenta; the features are represented by blue triangles and the ellipses of uncertainty associated with each feature are in solid red.

Figure 8 shows the map reconstruction based on the EKF-SLAM with the maximum eigenvalue feature selection criterion presented en Section 4.3. Figure 8(a) shows the case when *LIM* = 5 and Figure 8(b) shows the case for *LIM* = 2.

**Figure 9.**
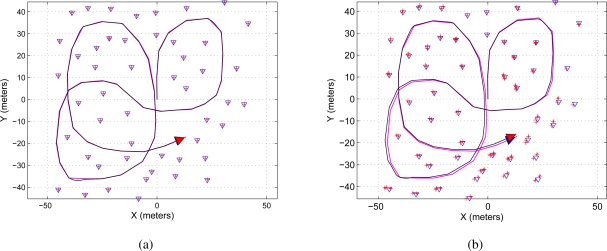
Map reconstruction of the environment using the features’ covariance selection criterion on the EKF-SLAM. **(a)** Shows the map reconstruction when the five most significant features—*LIM* = 5 in Algorithm 5—were used for the correction stage of the EKF-SLAM; **(b)** shows the map reconstruction when *LIM* = 2. The path traveled by the mobile robot is represented in a solid black line and the path estimated by the SLAM in solid magenta; the features are represented by blue triangles and the ellipses of uncertainty associated with each feature are in solid red.

Finally, Figure 9 shows the map reconstruction based on the EKF-SLAM with the *features’ covariance* approach. Figure 9(a) shows the case when *LIM* = 5 and Figure 9(b) shows the case for *LIM* = 2. As it can be seen, the results shown in Figure 9 are very similar to the ones shown in Figure 6.

**Figure 10.**
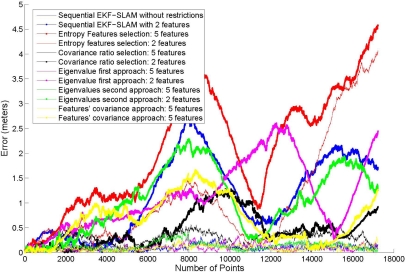
Evolution of the error of the different estimated paths by the EKF-SLAM algorithms proposed herein with respect to the predefined path. As it can be seen, the path estimated by the EKF-SLAM with the feature selection approach based on the covariance ratio shows the closest path to the one estimated by the full sequential EKF-SLAM. The path estimated by the entropy approach shows the worst path (with the higher error evolution). The *Number of points* axis refers to the successively points of the path used for the calculation of the error.

**Figure 11.**
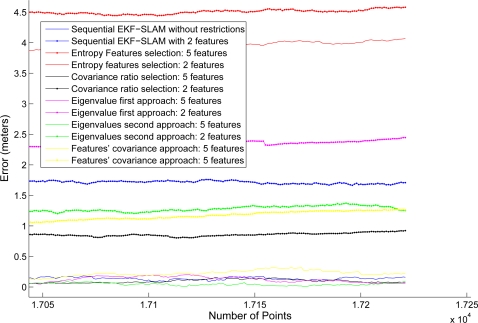
Zoom of Figure 10. As it can be seen, the paths estimated by the entropy approach is the worst estimated path.

For the purpose of showing the performance of each SLAM algorithm, Table 1 shows the mean square error (MSE) between the pre-established path and the estimated path by the SLAM algorithms. The MSE associated with each algorithm was calculated point-to-point according to the data stored from the mobile robot pose SLAM estimation and the predefined path. In addition, Figure 10 shows the error evolution between the estimated path and the predefined one. As it can be seen, the full sequential EKF-SLAM shows the smallest error at all time whereas the EKF-SLAM with the feature selection criterion based on the covariance ratio shows the closest evolution with respect to the full sequential EKF-SLAM. Furthermore, the EKF-SLAM with the feature selection method based on the entropy approach shows the worst path between all the executions. Figure 11 shows a zoom of Figure 10.

As it is shown in Table 1, when increasing the number of features to be used within the correction stage, decreases the MSE associated with the path estimated by the SLAM. Among the five feature selection approaches presented in this work, the feature selection criterion based on the covariance ratio approach has shown the best performance with both: *LIM* = 5 and *LIM* = 2. The entropy selection approach has shown to be the worst criterion given the experiment shown in Figure 5. Furthermore, the covariance ratio approach and both eigenvalues approaches have shown a better MSE when *LIM* = 2 than the sequential EKF-SLAM with the correction of the two first detected features (see Figure 4(b)). In addition, at the end of the experiments shown in Figures 4–9, the size of the map was of 50 point-based features for the full sequential EKF-SLAM—with no feature selection; for the EKF-SLAM with the entropy feature selection approach restricted to the two most significant features, the SLAM’s map was of 150 features—mainly because of both: the bad association given by the Mahalanobis distance criterion used in this work [3] and the increasing processing time; the map obtained by the EKF-SLAM with the covariance ratio feature criterion approach restricted to two features was of 58 point-features whereas the map obtained by the EKF-SLAM with sum of eigenvalues and the maximum eigenvalue feature selection approaches restricted to two features was of 68 and 65 point-based features respectively. For the features’ covariance approach, the number of features detected was of 60. As it can be seen, the EKF-SLAM with the covariance ratio feature selection approach and the features’ covariance approach show the minimum map reconstruction when compared with the other methods with feature selection restriction.

The implementation results shown in Figures 6–8 were carried out using the Joseph’s covariance matrix instead of the classical SLAM covariance matrix correction shown in Equation (1). The results of using the covariance matrix correction shown in Equation (1) have not shown significant map reconstruction differences with respect to the Joseph’s approach. Hence, the graphical results were not included herein.

### 5.2. Feature’s Covariance Evolution

**Figure 12.**
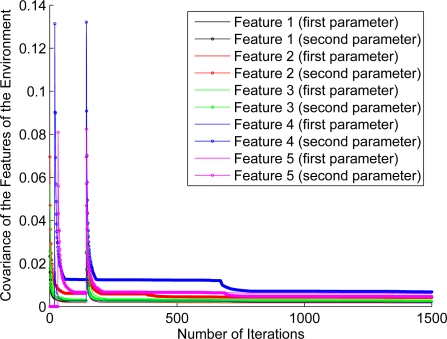
Covariance evolution of a set of five features—with two parameters—according to the estimation carried out by the full EKF-SLAM without feature restriction.

**Figure 13.**
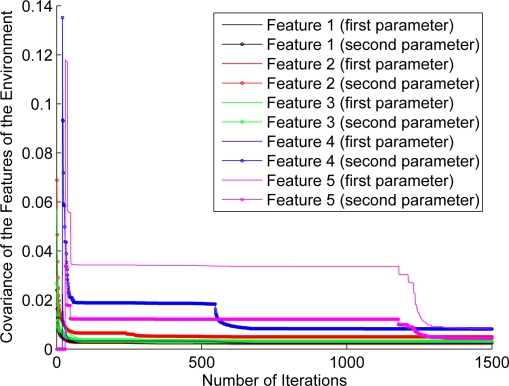
Covariance evolution of a same set of features of Figure 12 estimated by EKF-SLAM with the entropy feature selection approach.

**Figure 14.**
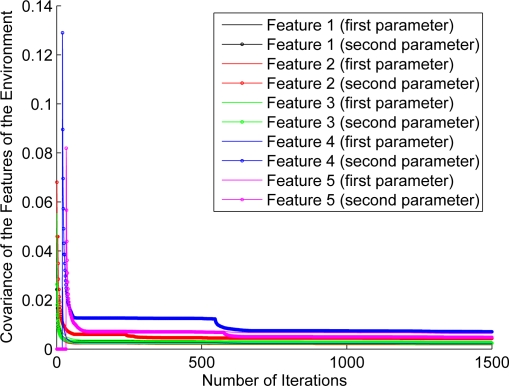
Covariance evolution of a same set of features of Figure 12 estimated by EKF-SLAM with the covariance ratio feature selection approach.

**Figure 15.**
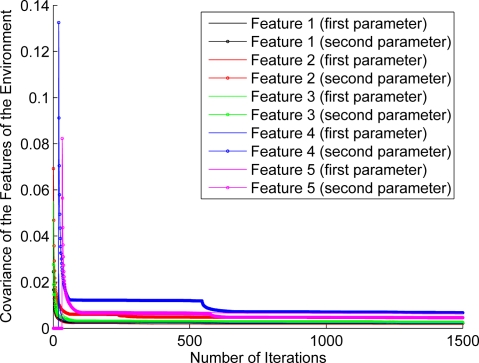
Covariance evolution of a same set of features of Figure 12 estimated by EKF-SLAM with the *sum of eigenvalues* feature selection approach.

**Figure 16.**
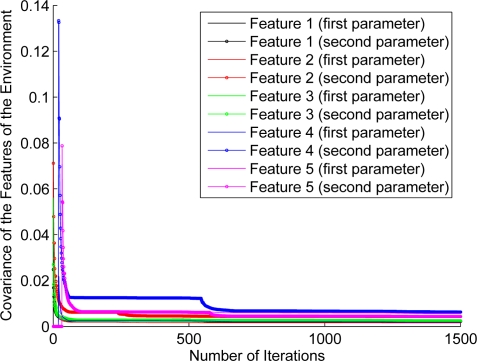
Covariance evolution of a same set of features of Figure 12 estimated by EKF-SLAM with the maximum eigenvalue feature selection approach.

**Figure 17.**
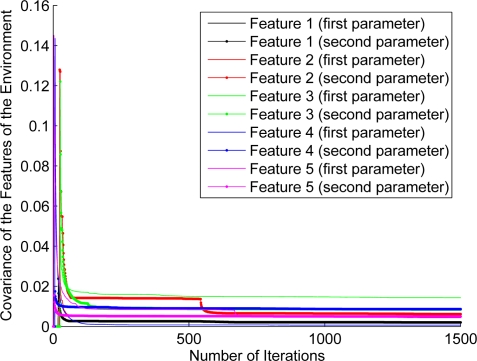
Covariance evolution of a same set of features of Figure 12 estimated by EKF-SLAM with the features’ covariance selection approach.

For the purpose of showing the evolution of the features’ estimation, Figures 12–17 show the evolution of the covariance of five features—with two parameters per feature—at different stages of the navigation of the experiments shown in Figures 4–9. Figure 12 shows the covariance evolution of the features when estimated by the full sequential EKF-SLAM without restrictions to the correction stage; Figure 13 shows the evolution of the same set of features when estimated by the EKF-SLAM with the entropy feature selection approach; Figure 14 shows the evolution of the features when using the EKF-SLAM with the covariance ratio feature selection approach implemented; Figures 15 and 16 show the evolution of such set of features when estimated by the EKF-SLAM with the sum of eigenvalues and the maximum eigenvalue selection approaches respectively. Figure 17 shows the feature covariance evolution for the features’ covariance approach. In Figures 13–17, the magenta feature and the green feature are not used in the correction stage. The last means that, although those features are added to the SLAM system state, they were not considered as most significant features at the moment of the correction of the SLAM algorithm. As it can be seen, there is no difference between the convergence of non-significant features compared with significant ones.

### 5.3. Processing Time

**Figure 18.**
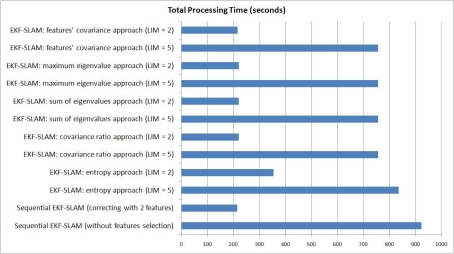
Accumulated processing time associated with each EKF-SLAM with feature selection criterion approach.

With the aim of showing the processing resources used by each EKF-SLAM with feature selection criterion algorithm, Figure 18 shows the accumulated processing time associated with them. Figure 18 represents the amount of time that each algorithm of Table 1 remained processing data. As expected, the algorithms with a feature selection criterion have shown a lower accumulated processing time than the ones with non restrictions in the correction stage of the EKF-SLAM. Furthermore, the EKF-SLAM with the feature selection criterion based on the entropy (Section 4.1) has a bigger amount of processing time when compared with the others algorithms—with the exception of the sequential EKF-SLAM with non feature selection criterion. The increment on its accumulated processing time is due to the determinant of the covariance matrix of the SLAM system state, as stated in the information calculation shown in Equations (3) and (4). Thus, as increases the number of elements on the SLAM system state, increases the computational cost of calculating the determinant of the covariance matrix associated with it.

The improvement of the restrictions within the sequential EKF-SLAM is linear with *O*(*n*^2^).

## 6. Conclusions

This paper has presented several non-heuristic feature selection criteria for an EKF-SLAM algorithm. The feature selection criteria were based on the convergence theorem of the EKF-SLAM. Thus, only the features that cause the highest improvement in the convergence of the covariance matrix determinant of the SLAM system state were chosen for the correction stage of the algorithm. These features were called as the most significant features.

Four feature selection approaches were shown in this work. The first approach consisted of selecting the most significant features based on the covariance ratio evaluation. The second approach was based on the sum of the eigenvalues and the third was based on maximum eigenvalue associated to (*I − K_t_H_t_*) in the correction stage of the EKF-SLAM. The fourth approach was based on the selection of features according to their covariance matrix and their meaning to the EKF-SLAM convergence. Furthermore, the feature selection proposals were extended for the case the Joseph’s covariance correction form were used instead of the classical expression of the correction stage of the SLAM’s covariance matrix. For all the approaches, the corresponding algorithms, the optimization criterion and the calculation reductions were also shown.

Each EKF-SLAM with the feature selection criterion was compared with the sequential EKF-SLAM (where no feature selection restrictions were available), with a sequential EKF-SLAM where only the first two features were used in the correction stage and with a feature selection procedure based on the entropy associated with the covariance matrix of the SLAM algorithm. Several experimental results were also carried out, showing the performance of each feature selection proposal.

Thus, for an outdoor environment composed by trees, the EKF-SLAM with feature selection criterion based on the covariance ratio and the features’ covariance approach have shown a better performance than the rest of the feature selection criteria, showing the lowest mean square error of the traveled path when using only the two most meaningful features and the smallest processing time. On the other hand, the entropy based selection has shown the highest mean square error. Also, the EKF-SLAM with the entropy based selection criterion was the algorithm with the highest computing demanding resources, mainly because of the determinant of the complete covariance matrix within its calculations.

Despite of the fact that in this work the EKF-SLAM algorithm was based on point-based features, the selection criteria proposed herein are independent of the kind of features used. Furthermore, the feature selection criteria can be combined in order to robust the feature selection procedure.

## Figures and Tables

**Table 1.** Mean Square Error associated with the different SLAM algorithms with feature selection criterion.

SLAM algorithms	MSE (*m*^2^)
Sequential EKF-SLAM (without feature selection)	0.11
Sequential EKF-SLAM (correcting with 2 features)	1.14
EKF-SLAM: entropy approach (*LIM* = 5)	1.09
EKF-SLAM: entropy approach (*LIM* = 2)	2.11
EKF-SLAM: covariance ratio approach (*LIM* = 5)	0.16
EKF-SLAM: covariance ratio approach (*LIM* = 2)	0.46
EKF-SLAM: sum of eigenvalues approach (*LIM* = 5)	0.15
EKF-SLAM: sum of eigenvalues approach (*LIM* = 2)	1.14
EKF-SLAM: maximum eigenvalue approach (*LIM* = 5)	0.17
EKF-SLAM: maximum eigenvalue approach (*LIM* = 2)	1.06
EKF-SLAM: features’ covariance approach (*LIM* = 5)	0.16
EKF-SLAM: features’ covariance approach (*LIM* = 2)	0.63
